# An aphid inspired metaheuristic optimization algorithm and its application to engineering

**DOI:** 10.1038/s41598-022-22170-8

**Published:** 2022-10-27

**Authors:** Renyun Liu, Ning Zhou, Yifei Yao, Fanhua Yu

**Affiliations:** 1 Department of Mathematics, Changchun Normal University, Changchun, 130032 Jilin China; 2 Department of Computer Science, Changchun Normal University, Changchun, 130032 Jilin China; 3 Department of Computer Science, Beihua University, Jilin, 132013 Jilin China

**Keywords:** Computational science, Computer science

## Abstract

The biologically inspired metaheuristic algorithm obtains the optimal solution by simulating the living habits or behavior characteristics of creatures in nature. It has been widely used in many fields. A new bio-inspired algorithm, Aphids Optimization Algorithm (AOA), is proposed in this paper. This algorithm simulates the foraging process of aphids with wings, including the generation of winged aphids, flight mood, and attack mood. Concurrently, the corresponding optimization models are presented according to the above phases. At the phase of the flight mood, according to the comprehensive influence of energy and the airflow, the individuals adaptively choose the flight mode to migrate; at the phase of attack mood, individuals use their sense of smell and vision to locate food sources for movement. Experiments on benchmark test functions and two classical engineering design problems, indicate that the desired AOA is more efficient than other metaheuristic algorithms.

## Introduction

Optimization has been widely used in engineering, aerospace, medical and many other fields^1^. Many optimization problems of practical as well as theoretical importance consist of searches for the “best” configuration of a set of variables to achieve optimal goals^2^. Currently, the prevailing methods for solving optimization problems are deterministic algorithms and metaheuristic algorithms. Although deterministic algorithms are efficient and useful in solving unimodal problems, most optimization problems are multimodal in the real world. For these problems, deterministic algorithms can easily fall into local optima^3^.This leads to the need for more reliable optimization techniques^4^. Metaheuristic algorithms are proposed to meet such a need. A metaheuristic algorithm is a stochastic optimization algorithm. As such, it combines a stochastic method with a local search method. Compared with deterministic algorithms, metaheuristic algorithms have attracted considerable attention owing to their efficiency^5^. Many deterministic optimization methods tend to fall into local optima easily because of the lack of randomness. In comparison, a metaheuristic algorithm uses randomness in local searches. As a result, it can effectively avoid being trapped in a local optimal solution.

Metaheuristic algorithms can be divided into two main categories: single solution-based and population-based^6^. The difference between the two approaches is that the population-based approach performs a complete calculation of the algorithm with a set of solutions rather than a single solution. The population-based approach, which simulates the behavior of groups by observing them and applying it to the optimization fields, can be further divided into four categories^7,8^: evolutionary-based algorithms, swarm intelligence-based algorithms, human-based algorithms, and physics and chemistry-based algorithms. Although these algorithms are classified differently, they have one thing in common: the search phase is divided into the exploration phase and the exploitation phase^9^ In the exploration phase, the algorithm explores multiple regions of the decision space benefitting from globality and randomness. The exploitation phase is usually carried out after the exploration phase is completed. At this phase, the algorithm pays more attention to the local area to improve the accuracy of the algorithm.

In recent years, metaheuristic algorithms have been widely used because of their reliability. However, according to the No Free Lunch (NFL) principle^10^, these metaheuristic algorithms cannot obtain global optimal solutions for all optimization problems. Inspired by the NFL principle, this study proposes a new algorithm named “Aphid Optimization Algorithms (AOA)” to broaden the investigation of metaheuristic optimization algorithms.

The basic idea of an AOA is to simulate aphids' behavior when searching for food, including the stage of winged aphids' production, flight mood, and attack mood. To the best of our knowledge, this is the first optimization algorithm based on the foraging behavior of aphids. In the generation phase, the clustering method is applied to simulate the generation of winged aphids; in the flight mood, the crowding state of the aphid population is simulated by introducing the crowding distance, and aphid individuals select their flight mode adaptively according to the current crowding state, enhancing the algorithm's exploration ability and diversity; in the attack mood, the optimal solution is guided to ensure accuracy. The main contributions of this paper can be summarized as follows:

- A new bio-inspired metaheuristic algorithm, the Aphid Optimization Algorithm, is proposed.
- The global search and local search are considered in the AOA to ensure the exploration and development capability of the algorithm.
- The effectiveness of the AOA is verified through simulation experiments and the solution of constrained engineering problems. These experiments demonstrate that AOA not only has the feature of high convergence accuracy but also has a fast convergence speed.

The rest of this paper is organized as follows: " Literature review" section reviews the related literature on population-based metaheuristic algorithms. In "Aphid optimization algorithm", aphid behavior and aphid optimization algorithm are introduced in detail. "Experimental results and discussion" Section  first presents and analyzes the experimental results of AOA for benchmark optimization problems. Then AOA is used to solve two classic engineering design problems. Finally, concluding remarks are presented in "Conclusion" section

## Literature review

**Figure 1:**
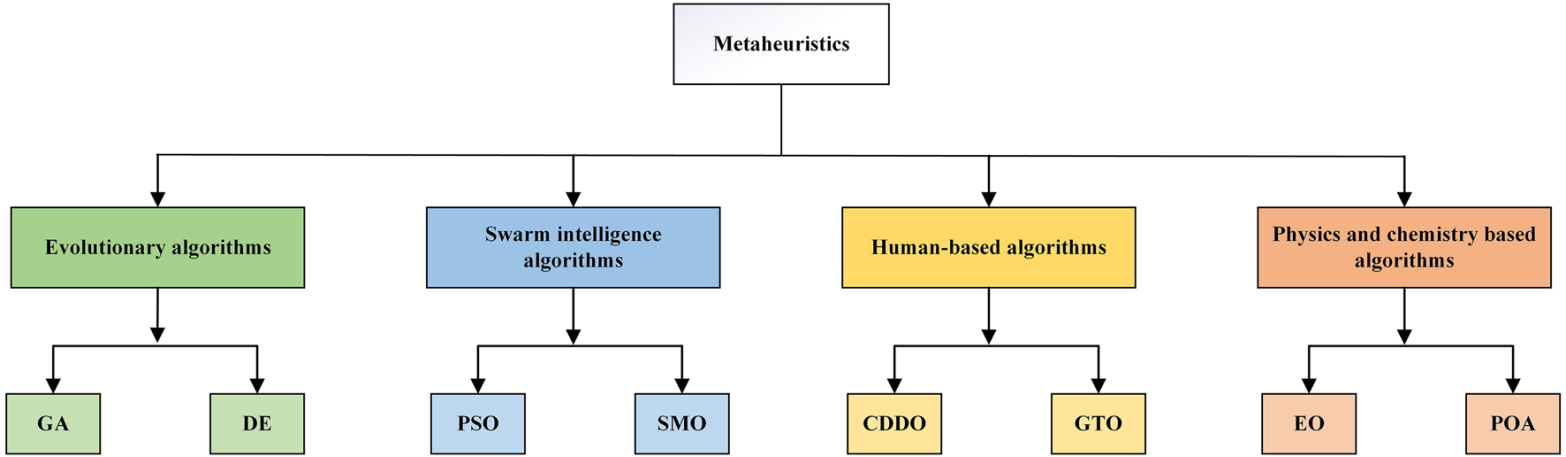
Classification of population-based metaheuristic algorithms**.** Four classes of population-based metaheuristics are introduced, and the corresponding more classical algorithms are cited.

Significant attention in the optimization field has been given to population-based optimization algorithms due to their simplicity and efficiency. Population-based algorithms can be classified into four categories: evolutionary-based algorithms, human-based algorithms, physics, and chemistry-based algorithms, and swarm intelligence-based algorithms (see Fig. 1).

Evolutionary-based algorithms are generally based on Darwin's evolutionary theory. Several popular evolutionary-based algorithms, such as the Genetic Algorithm (GA)^11^ and Differential Evolution (DE) algorithm^12^ have been proved to be highly efficient. GA uses the methods of reproduction, mutation, and selection among individuals in the population to exchange information for the optimal solution. In contrast, for DE, the mutation vector is generated by mutating the difference vector, which is crossed with the parental individuals to generate a new individual. This new individual is directly compared with the parental individual.

Human-based algorithm simulate many aspects of human behavior. Child Drawing Development Optimization (CDDO)^13^ is among the powerful human-based algorithms proposed recently. The motivation of CDDO is children's learning behavior and cognitive development in early childhood. Group Teaching Optimization (GTO)^14^, Poor and Rich Optimization Algorithm (PRO)^15^, Supply-Demand-Based Optimization (SDO)^16^, Search and Rescue Optimization Algorithm(RSO)^17^, and Student Psychology Based Optimization Algorithm (SPBO)^18^ are other popular examples of human-based algorithms.

Physics and Chemistry-based algorithms are inspired by chemical reaction phenomena or laws of physical phenomena. For example, Equilibrium Optimizer (EO)^19^ is a new optimization algorithm inspired by the control volume mass balance model, which is used to estimate the dynamic and equilibrium state. Billiards-inspired Optimization Algorithm (BOA)^20^, Big-Bang Big-Crunch(BB-BC)^21^, Central Force Optimization (CFO)^22^, Electron Radar Search (ERS) algorithm^23^, Gravitational Search (GS) algorithm^24^, and Planet Optimization Algorithm (POA)^25^ are also representatives of this kind of algorithms.

Swarm intelligence-based algorithms originate from simulations of biological group behavior or foraging behavior in nature. In such an algorithm, the search and optimization process is simulated as evolving or foraging process to solve optimization problems. Particle Swarm Optimization (PSO)^26^, one of the most popular swarm intelligence-based algorithms, is inspired by birds foraging. The algorithm gives the position and velocity of each particle, and each particle updates its position by updating its velocity. Through iterative search, the population can constantly find better positions to get optimal solutions for optimization. Butterfly Optimization Algorithm (BOA)^27^ solves optimization problems based on the foraging process of butterflies. Butterflies use their sensors to locate the food source. In BOA, it is assumed that each butterfly produces a scent of a certain intensity, which is transmitted and sensed by other butterflies in the area. Harris Hawks Optimization (HHO) algorithm^28^ finds the optimal solution by simulating the predation behavior of Harris Hawks. According to the current state of the prey, Harris Hawks choose different attack methods to hunt. Slime Mould Algorithm (SMA)^29^ simulates the diffusion and foraging behavior of slime mold. It uses adaptive weights to simulate the process of positive and negative feedback generated by the propagation wave based on biological oscillators, forming an optimal path to connect food. Additionally, some scholars have proposed other effective excellent algorithms, such as, Fitness Dependent Optimizer (FDO)^30^, Ebola Optimization Search Algorithm(EOSA)^31^, Aquila Optimizer(AO)^32^, Farmland fertility^33^, Barnacles Mating Optimizer (BMO)^34^, and Falcon Optimization Algorithm(FOA)^35^.

Despite their abilities to solve optimization problems, existing optimization methods face challenges. For instance, the accuracy of GA^11^ needs to be improved; DE^12^ tends to fall into a local optimum; and PSO^26^ and BOA^27^ may converge prematurely, resulting in the decline of population diversity. In this article, to resolve some of the shortcomings of the above optimization algorithms, we propose the Aphid Optimization Algorithm (AOA). The algorithm simulates aphids' foraging behavior while taking into account natural environmental factors, such as density, temperature, light, and wind. By designing a mechanism for adaptively selecting flight mode, the diversity of the population is increased and the exploration ability of the algorithm is improved. At the same time, using the optimal solution to guide individuals' movement could ensure the algorithm's accuracy.

## Aphid optimization algorithm

In this section, the general biology of the aphid and the optimization model of the proposed algorithm will be discussed in detail.

### Biological habits

Aphids are herbivorous insects, which are widely distributed around the world, but mainly concentrated in temperate regions. About 4700 species of aphids have been discovered^36^. Like other species of migratory insects, many species of aphids possess the nature of migration. Migration allows aphids to successfully avoid adverse environments to find appropriate host plants for feeding, and the winged aphid is the prerequisite for aphid migration. The generation of winged aphids is influenced by many factors, including crowded environment, temperature, light, and so on^37^. As a result, pheromones will be produced among populations to stimulate the production of winged aphids to migrate.

Aphids in the flight stage can be divided into active flight and passive flight according to the nature of flight. Active flight is the spiral flight under the control of the aphid itself, while passive flight is the linear flight driven by the wind^38^. Such uninterrupted flight will cause the winged aphid's energy reserves to be constantly depleted. When the stored energy is low, the aphid cannot fly on its own but relies on the wind to migrate. When there is more energy of its own, the aphid power system and resistance are stronger, and individuals perform the active flight. At this time, when the population is more crowded, the poorer survival conditions cause individuals to migrate to sparse areas. With the increase in flight time, the aphids are inclined to the long-wave light of the hosts and enter the attack stage.

In the attack stage, the interaction of smell and vision lures individuals to land and find hosts to feed on. Winged aphids search for their hosts, mainly by identifying host-specific volatiles through olfactory sensors. The individual's sense of smell navigates its landing based on the odor of the host plant. At the same time, the yellow wave light in the host during flight is a strong stimulus to the visual organs of winged aphids^39^. Therefore, in locating the host location, the visual signal reflected from the host has a solid attraction to aphids. In summary, the foraging process of aphids can be broadly summarized in the following three stages: the production of winged aphids, migration, and the search for hosts to feed.

### Mathematical model

**Figure 2:**
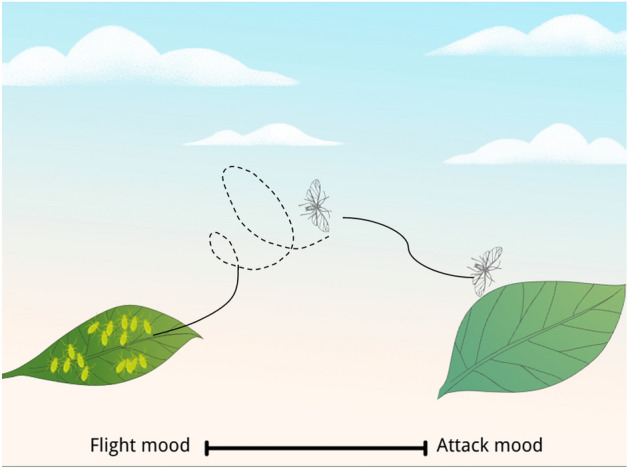
Process of flight mood and attack mood. The simulation of aphid migration and attack.

Kaveh proposed the Cyclical Parthenogenesis Algorithm (CPA)^40^, which was inspired by the reproduction behavior of aphids. In the algorithm, some rules are enforced according to the life cycle of aphids, and it iteratively updates the solutions with these rules followed. The aphid optimization algorithm proposed in this paper is inspired by the whole process of aphid foraging, including the generation phase of the winged aphids, the phase when winged aphids migrate in order to find the host, and the phase when winged aphids locate the host location to feed. We call these three phases generation stage, flight mood, and attack mood. According to the process of foraging, we will establish the mathematical model for each of the three phases. The whole process of flight and attack moods is shown in Fig. 2. The pseudo code of the entire process of AOA is shown in Algorithm 1.
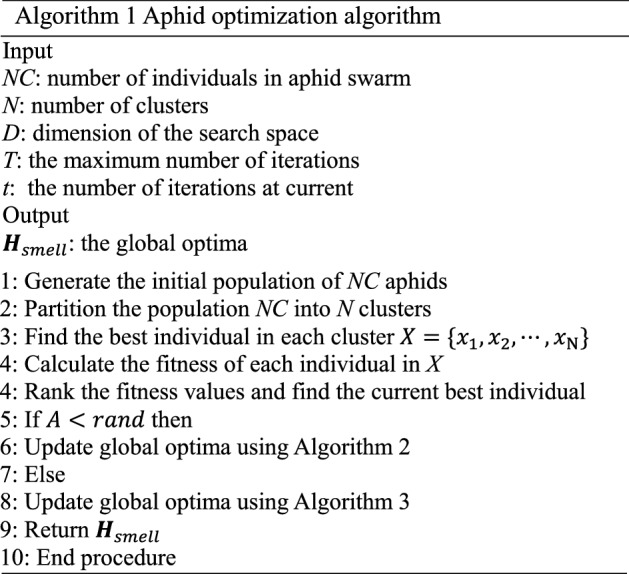

### Generation stage

Before taking off, individuals need to evolve into winged aphids in order to fly. However, not all aphids can evolve into winged aphids because of the influence of temperature, light, and crowding between populations. At the same time, aphids are not evenly distributed in their habitats but clustered in multiple locations. In order to simulate this stage, we adopt the method of K-means clustering^41^.

First, we randomly initialize to generate *NC* individuals, and perform K-means clustering on these individuals to generate *N* classes. In each class, according to the fitness value, the optimal individual is selected as the winged aphid for the next evolutionary operation.

### Flight mood

During the flight mood, the individuals begin the exploration phase. With the increase in flight time, the energy stored by the individuals gradually decreases. This process can be described mathematically as:

1 $$ E = 1 - \frac{t}{T} $$

where *E* denotes the energy of the aphid. The influence of wind as the driving force for aphids to fly cannot be underestimated. The effect of wind in this paper is represented as *w*, which can be simulated as a random number drawn from the random variable uniformly distributed in (0, 1) because the wind is randomly varying.

When the energy stored in an individual is relatively small, that is, when $E/w < 1$, aphids perform the passive flight. In this situation, the aphids fly in a straight line according to the direction of the wind. This process is simulated as:

2 $$ {\varvec{X}}_{i} (t + 1) = w*({\varvec{X}}_{n} (t) - {\varvec{X}}_{i} (t)) $$

where ${\varvec{X}}_{n}$ is a randomly selected individual in the population and ${\varvec{X}}_{i}$ denotes the position of the $i^{{{th}}}$ individual in the current population. When the individual’s stored energy is large, that is, when $E/w \ge 1$, the aphids fly actively. At this time, the aphids perform a spiral flight under their control. In this paper, the crowding distance $CD({\varvec{X}}_{i} )$ is used to judge the crowding degree of the individual, which can be denoted as follows:

3 $$ CD({\varvec{X}}_{i} ) = \sum\limits_{j = 1}^{N} {d_{i,j} } $$

where $CD({\varvec{X}}_{i} )$ represents the Euclidean distance from the $i\,{{{th}}}$ individual to the $j\,{{{th}}}$ individual in the population. The smaller the crowding distance is, the more crowded the current individual is. When the crowding distance is less than the average crowding distance $CD({\varvec{X}}_{avg} )$ between the population, it indicates that the individual is in a more crowded state; otherwise, it indicates that the individual is in a relatively loose state.

At this time, considering that the crowded environment is not conducive to the evolution of aphids, individuals will adaptively select appropriate individuals to guide them to spiral flight according to the crowding distance. Therefore, when the individual is in a crowded state, the individual with the largest crowding distance is selected to guide the current solution to the loose area. Otherwise, the crowd chooses a random individual to guide the flight. This process is simulated as:

4 $$ {\varvec{X}}_{i} (t + 1) = {\varvec{X}}_{i} (t) + w * ({e}^{R} \cos (2\uppi R) * ({\varvec{X}}_{m} (t) - {\varvec{X}}_{i} (t))) + w * ({e}^{R} \sin (2\uppi R) * ({\varvec{X}}_{m} (t) - {\varvec{X}}_{i} (t))) \;if\;CD({\varvec{X}}_{i} ) < CD({\varvec{X}}_{avg} ) $$

5 $$ {\varvec{X}}_{i} (t + 1) = {\varvec{X}}_{i} (t) + w * ({e}^{R} \cos (2\uppi R) * ({\varvec{X}}_{n} (t) - {\varvec{X}}_{i} (t))) + w * ({e}^{R} \sin (2\uppi R) * ({\varvec{X}}_{n} (t) - {\varvec{X}}_{i} (t)))\;else $$

**Figure 3:**
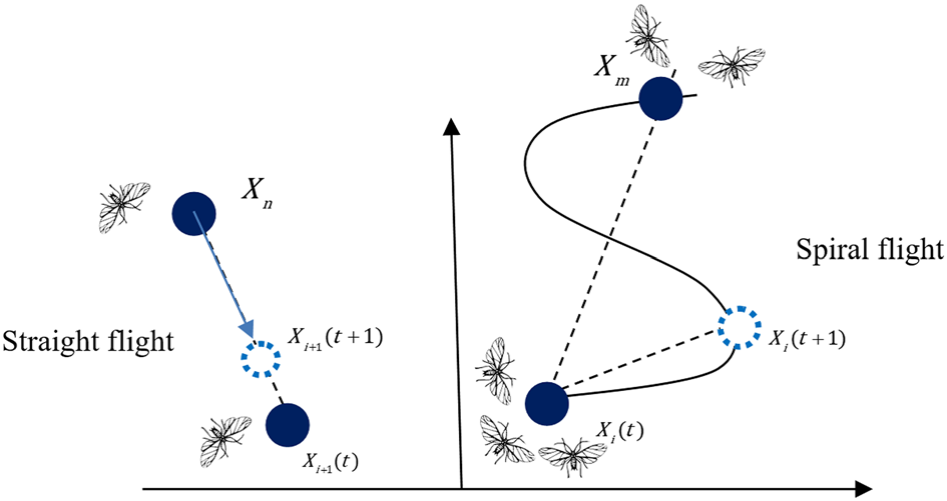
Movement of aphids in flight mood. Simulation of straight flight and spiral flight of aphid flight phase.

Here ${\varvec{X}}_{m}$ is the individual with the largest crowding distance in the current population and *R* is the spiral radius which is a uniformly distributed random variable in (−1, 1). Figure 3 depicts the movement of aphids in flight mood. The pseudo code of flight mood of AOA is shown in Algorithm 2.
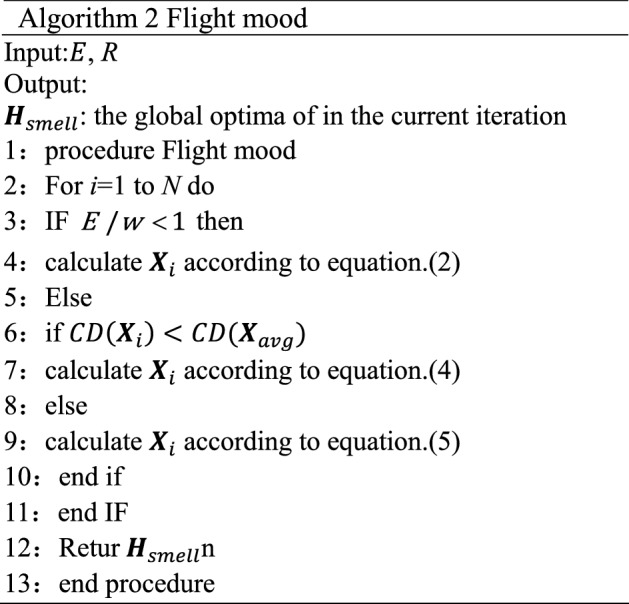

### Attack mood

**Figure 4:**
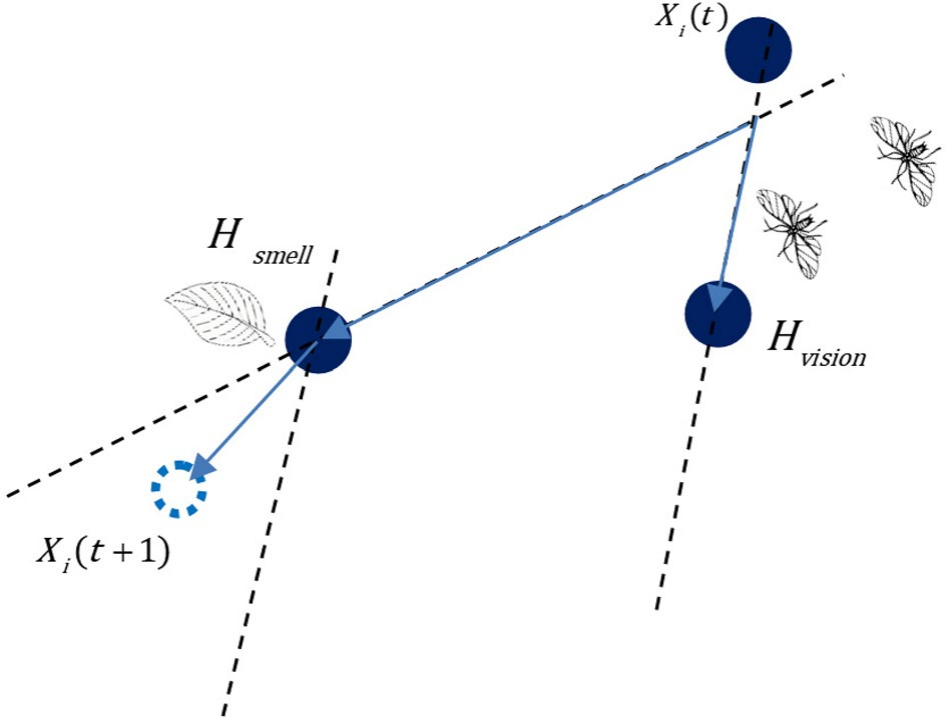
Movement of aphids in attack mood. Simulation of aphid’s movement in attack phase.

In the attack mood, individuals begin the exploitation phase. After entering the attack mood, the aphid will find its host, and it mainly navigates landing position by olfactory sense according to the smell of host plants, so the volatiles of host plants play an important role in the tendency of the aphid to fly to its host. Simultaneously, the visual sense is also a significant player in the search for the host. The long wave light of the host plant will affect the landing of aphids because aphids have a strong tendency to the long wave light. The location of host plants by smell ${\varvec{H}}_{smell}$ is regarded as the global optimal solution, and the location of host plants by vision ${\varvec{H}}_{vision}$ is regarded as the personal optimal location. Figure 4 shows the movement of aphids in an attack mood. This process is simulated as:

6 $$ {\varvec{X}}_{i} (t + 1) = {\varvec{H}}_{smell} (t) + {r} * (({\varvec{H}}_{smell} (t) - {\varvec{X}}_{i} (t)) + ({\varvec{H}}_{vision} (t) - {\varvec{X}}_{i} (t))) $$

where *r* is a random number between (0, 0.1). The pseudo code of Flight mood of AOA is shown in Algorithm 3.
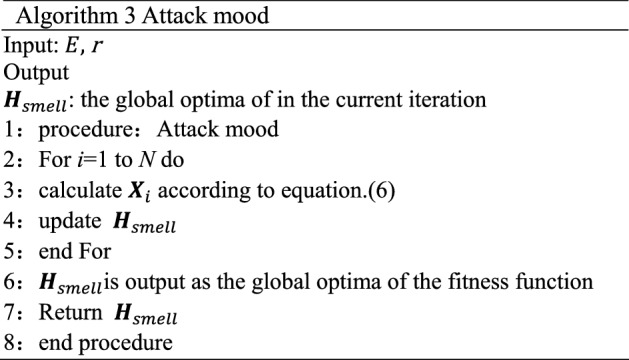

### Transition

Considering that aphids are stimulated by yellow waves during flight, the stimulation degree by yellow waves is simulated as follows:

7 $$ I = \frac{1}{{1 + {e}^{{ - \frac{1}{S + 1}}} }} $$

where *S* is the absolute value of Euclidean distance between the average position of the population and the host plant. The closer the distance, the greater the degree of stimulation and the value of *I*.

Aphids have a tendency to long-wave light from plants in the process of flight, so they enter the attack mood. We use the following formula to express the tendency of individuals to long-wave light:

8 $$ A = I * \left( {1 - \frac{t}{T}} \right) $$

Let *rand* be a number drawn from the uniformly distributed random variable in (0, 1). Then we can determine if the flight mood or the attack mood is performed as follows: when *A* < *rand*, the flight mood is performed; otherwise, the attack mood is performed.

## Experimental results and discussion

In order to verify the efficiency of the proposed algorithm, we compare the performance of the proposed algorithm with that of other algorithms. From the current popular metaheuristic algorithms, we choose six of them as comparison objects: PSO, DE, GA, BOA, SMA, HHO, MFO^42^, COA^43^, and CAMES^44^. The parameter setting of the above algorithms is shown in Table 1. In this section, we compare the proposed algorithm with other algorithms on 23 well-known benchmark test functions from the benchmark set proposed in^45^ and 10 benchmark test functions from CEC 2019. The constrained industrial optimization applications are also employed to demonstrate the efficiency of the proposed algorithm. All experiments are run with MATLAB 2018a. The details of the running system are shown in Table 2.

**Table 1:** Parameter setting of various optimization algorithms. The specific parameter settings of the algorithms compared in this paper.

Algorithm	Parameters	Value
PSO	Learning factors *C*_*1*_	2
	Learning factors *C*_*2*_	2
	Inertia weight	0.798
DE	Scaling factor	0.8
	Crossover constant	0.2
GA	Crossover probability	0.8
	Mutation probability	0.1
BOA	*p*	0.8
	power exponent	0.1
SMA	*z*	0.03
HHO	*β*	1.5
MFO	helix parameters *b*	1
COA	*N*_*pack*_	10
	*N*_*coyoye*_	5
AOA	*NC*	1000
	*N*	50

**Table 2:** System and resource characteristics. The running environment in this study.

System information	Content
Operating system	Windows 10
Processor	Intel(R) Core(TM) i5-7200U
CPU	@ 2.50 GHz(4 CPUs), ~ 2.7 GHz
RAM	8192 MB

To clearly compare the algorithm performance, in this paper, the maximum number of iterations is set to be 1000, and each function is repeated 30 times. The population size of the above algorithms is designed to be 50. The average values of the convergence results of 30 runs are calculated as an evaluation metric of the accuracy of the algorithm. The smaller the average value, the higher the convergence accuracy of the algorithm. Meanwhile, the convergence speed of the algorithm is compared by plotting the convergence curves.

### Benchmark function validation

We test the performance of the proposed AOA on the proposed benchmark sets. These 23 well-known functions are characterized as multimodal and unimodal as shown in Table 3. Additional new test functions of CEC 2019 are shown in Table 4. Here Type denotes the type of the function such as M: Multimodal and U: Unimodal, Dim represents the dimension of function, Range denotes the definition domain of the function, and Optima denotes the optimal value of the function. The simulation results of AOA on benchmark functions used in this study are shown in Table 5.

**Table 3:** Standard Benchmark functions. Specific 23 standard test functions, including the dimension, upper and lower bounds, and optimal value of each function.

No	Type	Formula	Dim	Range	Optima
F1	U	$\mathrm{f}(x)=\sum_{\mathrm{i}=1}^{\mathrm{n}}{{x}_{\mathrm{i}}}^{2}$	30	[−100,100]	0
F2	U	$\mathrm{f}(x)=\sum_{\mathrm{i}=1}^{\mathrm{n}}\left|{x}_{\mathrm{i}}\right|+\prod_{\mathrm{i}=1}^{\mathrm{n}}\left|{x}_{\mathrm{i}}\right|$	30	[−10,10]	0
F3	U	$\mathrm{f}(x)=\sum_{\mathrm{i}=1}^{\mathrm{n}}{(\sum_{\mathrm{j}=1}^{\mathrm{i}}{x}_{\mathrm{j}})}^{2}$	30	[−100,100]	0
F4	U	$\mathrm{f}(x)=\mathrm{max}(\left|{x}_{\mathrm{i}}\right|,1\le \mathrm{i}\le \mathrm{n})$	30	[−100,100]	0
F5	U	${\text{f}}\left( x \right) = \mathop \sum \limits_{{{\text{i}} = 1}}^{{{\text{n}} - 1}} \left[ {100\left( {x_{{{\text{i}} + 1}} - {\text{x}}_{{\text{i}}} ^{2} } \right)^{2} + \left( {x_{{\text{i}}} - 1} \right)^{2} } \right]$	30	[−30,30]	0
F6	U	${\text{f}}\left( x \right) = \mathop \sum \limits_{{{\text{i}} = 1}}^{{\text{n}}} \left( {x_{{\text{i}}} + 0.5} \right)^{2}$	30	[−100,100]	0
F7	U	$\mathrm{f}(x)= \sum_{\mathrm{i}=1}^{\mathrm{n}}\mathrm{i}{{x}_{\mathrm{i}}}^{4}+\mathrm{rand}(\mathrm{0,1})$	30	[−1.28,1.28]	0
F8	M	$\mathrm{f}(x)=-\sum_{\mathrm{i}=1}^{\mathrm{n}}\left[{x}_{\mathrm{i}}\mathrm{sin}(\sqrt{\left|{x}_{\mathrm{i}}\right|})\right]$	30	[−500,500]	−12,569.5
F9	M	$\mathrm{f}(x)=\sum_{\mathrm{i}=1}^{\mathrm{n}}\left({{x}_{\mathrm{i}}}^{2}-10\mathrm{cos}(2\uppi {x}_{\mathrm{i}})+10\right)$	30	[−5.12,5.12]	0
F10	M	$\mathrm{f}(x)=-20\mathrm{exp}(-0.2\times \sqrt{\frac{1}{\mathrm{n}}\sum_{\mathrm{i}=1}^{\mathrm{n}}{{x}_{\mathrm{i}}}^{2}})-\mathrm{exp}(\frac{1}{\mathrm{n}}\sum_{\mathrm{i}=1}^{\mathrm{n}}\mathrm{cos}(2\uppi {x}_{\mathrm{i}}))+20+\mathrm{exp}(1)$	30	[−32,32]	0
F11	M	$\mathrm{f}(x)=\frac{1}{4000}\sum_{\mathrm{i}=1}^{\mathrm{n}}{{x}_{\mathrm{i}}}^{2}-\prod_{\mathrm{i}=1}^{\mathrm{n}}\mathrm{cos}\left(\frac{{x}_{\mathrm{i}}}{\sqrt{\mathrm{i}}}\right)+1$	30	[−600,600]	0
F12	M	$\mathrm{f}(x)=\frac{\pi }{n}\left\{10{sin}^{2}\left(\pi {y}_{i}\right)+{\sum }_{i=1}^{n-1}{\left({y}_{i}-1\right)}^{2}[1+10{sin}^{2}(\pi {y}_{i+1})]+{{(y}_{n}-1)}^{2}\right\}+{\sum }_{i=1}^{n}u({x}_{i},\mathrm{10,100,4})$	30	[−50,50]	0
F13	M	$\mathrm{f}(x)={(1.5-{x}_{1}+{x}_{1}{x}_{2})}^{2}+{(2.25-{x}_{1}+{x}_{1}{{x}_{2}}^{2})}^{2}+{(2.625-{x}_{1}+{x}_{1}{{x}_{2}}^{3})}^{2}$	2	[−4.5,4.5]	0
F14	M	$\mathrm{f}(x)={\left(\frac{1}{500}+\sum_{\mathrm{j}=1}^{25}\frac{1}{j+{\left({x}_{i}-{a}_{ij}\right)}^{6}}\right)}^{-1}$	2	[−65.536,65.536]	1
F15	M	$\mathrm{f}(x)=\sum_{\mathrm{i}=1}^{11}{\left[{a}_{i}-\frac{{x}_{1}\left({{b}_{i}}^{2}+{b}_{i}{x}_{2}\right)}{{{b}_{i}}^{2}+{b}_{i}{x}_{3}+{x}_{4}}\right]}^{2}$	4	[−5,5]	0.00030
F16	M	$\mathrm{f}\left(x\right)={{4x}_{1}}^{2}-2.1{{x}_{2}}^{4}+\frac{1}{3}{{x}_{1}}^{6}+{x}_{1}{x}_{2}-4{{x}_{2}}^{2}+4{{x}_{2}}^{4}$	2	[−5,5]	−1.0316
F17	M	$\mathrm{f}\left(x\right)={\left({x}_{2}-\frac{5.1}{4{\pi }^{2}}{{x}_{1}}^{2}+\frac{5}{\pi }{x}_{1}-6\right)}^{2}+10\left(1-\frac{1}{8\pi }\right)\mathrm{cos}{x}_{1}+10$	2	[−5,10]×[0,15]	0.398
F18	M	$\mathrm{f}(x)=\left[1+{\left({x}_{1}+{x}_{2}+1\right)}^{2}(19-14{x}_{1}+3{{x}_{1}}^{2}-14{x}_{2}+6{x}_{1}{x}_{2}+3{{x}_{2}}^{2})\right]\times \left[30+{\left(2{x}_{1}-3{x}_{2}\right)}^{2}\times \left(18-32{x}_{1}+12{{x}_{1}}^{2}+48{x}_{2}-36{x}_{1}{x}_{2}+27{{x}_{2}}^{2}\right)\right]$	2	[−2,2]	3
F19	M	$\mathrm{f}\left(x\right)=-\sum_{i=1}^{4}{c}_{i}exp{\left(-\sum_{j=1}^{4}{a}_{ij}\left({x}_{j}-{p}_{ij}\right)\right)}^{2}$	4	[0,1]	−3.86
F20	M	$\mathrm{f}\left(x\right)=-\sum_{i=1}^{4}{c}_{i}exp{\left(-\sum_{j=1}^{6}{a}_{ij}\left({x}_{j}-{p}_{ij}\right)\right)}^{2}$	6	[0,1]	−3.32
F21	M	$\mathrm{f}\left(x\right)=-\sum_{i=1}^{5}{\left[\left(x-{a}_{i}\right){\left(x-{a}_{i}\right)}^{T}+{c}_{i}\right]}^{-1}$	4	[0,10]	−10.1532
F22	M	$\mathrm{f}(x)=-\sum_{i=1}^{7}{\left[\left(x-{a}_{i}\right){\left(x-{a}_{i}\right)}^{T}+{c}_{i}\right]}^{-1}$	4	[0,10]	−10.4028
F23	M	$\mathrm{f}(x)=-\sum_{i=1}^{10}{\left[\left(x-{a}_{i}\right){\left(x-{a}_{i}\right)}^{T}+{c}_{i}\right]}^{-1}$	4	[0,10]	−10.5363

**Table 4:** CEC 2019 benchmark functions. Specific CEC2019 test functions, including the dimension, upper and lower bounds, and optimal value of each function.

No	Function	Dim	Range	Optima
cec01	Storn's chebyshev polynomial fitting problem	9	[−8192, 8192]	1
cec02	Inverse hilbert matrix problem	16	[−16384, 16384]	1
cec03	Lennard-jones minimum energy cluster	18	[−4, 4]	1
cec04	Rastrigin's function	10	[−100, 100]	1
cec05	Griewangk's function	10	[−100, 100]	1
cec06	Weierstrass Function	10	[−100, 100]	1
cec07	Modified schwefel's function	10	[−100, 100]	1
cec08	Expanded schaffer's f6 function	10	[−100, 100]	1
cec09	Happy cat function	10	[−100, 100]	1
cec10	Ackley function	10	[−100, 100]	1

**Table 5:** Simulation results of AOA. Results of the proposed algorithm on each test function, including best solution, worst solution, average solution, and standard deviation.

Function	Aphid optimization algorithm			
	Mean	SD	Best	Worst
F1	0.00E + 00	0.00E + 00	0.00E + 00	0.00E + 00
F2	0.00E + 00	0.00E + 00	0.00E + 00	0.00E + 00
F3	0.00E + 00	0.00E + 00	0.00E + 00	0.00E + 00
F4	0.00E + 00	0.00E + 00	0.00E + 00	0.00E + 00
F5	1.81E−29	2.63E−02	2.77E−32	3.64E−25
F6	1.22E−26	5.92E−30	7.35E−28	8.42E−24
F7	1.57E−02	2.41E−02	4.93E−04	4.42E−02
F8	−1.26E + 04	4.12E−02	−1.26E + 04	−1.26E + 04
F9	0.00E + 00	0.00E + 00	0.00E + 00	0.00E + 00
F10	8.88E−16	0.00E + 00	8.88E−16	8.88E−16
F11	0.00E + 00	0.00E + 00	0.00E + 00	0.00E + 00
F12	2.17E−30	4.59E−31	6.25E−31	3.63E−28
F13	1.60E−30	2.09E−30	1.03E−30	5.21E−29
F14	9.98E−01	0.00E + 00	9.98E−01	9.98E−01
F15	1.74E−03	1.12E−04	8.63E−04	9.77E−02
F16	−1.03E + 00	0.00E + 00	−1.03E + 00	−1.03E + 00
F17	3.98E−01	0.00E + 00	3.98E−01	3.98E−01
F18	3.00E + 00	7.26E−08	3.00E + 00	3.00E + 00
F19	−3.86E + 00	3.58E−11	−3.86E + 00	−3.86E + 00
F20	−2.71E + 00	1.75E−01	−3.32E + 00	−2.56E + 00
F21	−1.01E + 01	1.87E−15	−1.01E + 01	−1.01E + 01
F22	−1.04E + 01	5.92E−16	−1.04E + 01	−1.04E + 01
F23	−1.05E + 01	1.54E−02	−1.05E + 01	−1.05E + 01
cec01	6.68E + 04	1.65E + 04	4.78E + 04	7.55E + 04
cec02	1.74E + 01	4.49E−01	1.75E + 01	1.74E + 01
cec03	1.27E + 01	6.87E−08	1.27E + 01	1.30E + 01
cec04	9.45E + 01	3.30E + 01	3.80E + 01	1.04E + 02
cec05	2.25E + 00	2.23E−01	2.11E + 00	2.84E + 00
cec06	9.08E + 00	7.81E−01	8.57E + 00	1.12E + 01
cec07	2.75E + 02	7.96E + 01	2.57E + 02	3.48E + 02
cec08	6.02E + 00	5.94E−01	5.96E + 00	7.46E + 00
cec09	3.15E + 00	4.67E−01	2.71E + 00	4.32E + 00
cec10	2.01E + 01	1.15E−01	2.01E + 01	2.03E + 01

### Analysis of simulation results

To compare AOA with other algorithms, the average and standards are listed in Table 6, where the mean in bold indicates optimal. We can see from the table that in most test functions, AOA performs better than other algorithms on both indicators: the average and standard deviation. From Table 6, it can be concluded that AOA obtains the best results on 20 out of 33 benchmark functions, ranking first in the number of best results among all algorithms. The second in the number of best results is SMA, which has 17 out of 33 benchmark functions. According to the results in Table 6, AOA algorithm is significantly superior in solving the unimodal test function and multimodal test functions.

**Table 6:** Comparison of AOA with different algorithms. The results of AOA with other different algorithms on each test function and the optimal solution is marked in bold. (significant values are in bold).

		AOA	DE	PSO	BOA	GA	SMA	HHO	MFO	COA	CMAES
F1	Mean	**0.00E + 00**	5.60E−03	1.64E−02	5.66E−10	1.55E + 01	6.55E−311	1.25E−194	4.12E−31	1.49E + 01	6.40E−62
	SD	0.00E + 00	2.10E−03	8.61E−03	1.58E−10	7.09E + 00	0.00E + 00	0.00E + 00	1.45E−30	9.74E + 00	5.29E−61
F2	Mean	**0.00E + 00**	8.15E−03	1.79E + 00	5.42E−18	1.27E + 00	9.68E−140	2.24E−100	4.32E−19	2.74E−01	1.98E−30
	SD	1.81E−149	1.35E−03	1.09E + 00	1.56E−15	2.45E−01	5.30E−139	1.23E−99	4.26E−09	1.15E−01	1.23E−30
F3	Mean	**0.00E + 00**	4.59E + 04	4.07E−01	5.14E−10	1.38E−03	8.85E−229	3.00E−160	1.56E−01	2.97E + 02	1.73E−41
	SD	0.00E + 00	9.24E + 03	2.48E−01	1.15E−10	2.60E−03	0.00E + 00	1.64E−159	6.84E−01	1.34E + 02	4.09E−41
F4	Mean	**0.00E + 00**	1.94E + 01	6.64E−01	4.46E−07	6.85E + 00	5.25E−176	4.57E−101	1.47E−06	1.38E + 01	5.03E−28
	SD	0.00E + 00	1.99E + 00	2.88E−01	7.97E−08	1.04E + 00	0.00E + 00	1.12E−100	6.15E−06	4.00E + 00	2.73E−28
F5	Mean	**1.81E−29**	4.34E + 02	6.36E + 01	2.99E + 01	1.47E + 04	2.32E−01	5.55E−06	6.66E + 02	6.96E + 02	7.06E−02
	SD	2.63E−02	8.81E + 01	5.08E + 01	2.19E−02	5.16E + 03	1.84E−01	1.91E−07	1.22E + 03	5.46E + 02	1.62E−02
F6	Mean	1.22E−26	5.39E−03	1.42E−02	4.78E + 00	1.48E + 01	6.90E−04	8.99E−06	9.21E−24	1.25E + 01	**0.00E + 00**
	SD	5.92E−30	1.24E−03	8.01E−03	4.11E−01	5.04E + 00	4.76E−04	2.33E−05	9.20E−26	8.01E + 00	0.00E + 00
F7	Mean	1.57E−02	6.07E−01	9.76E−02	8.08E−04	3.82E−02	4.44E−02	**4.33E−05**	3.71E−02	3.88E−02	2.20E−03
	SD	2.41E−02	1.12E−01	1.70E−02	3.91E−04	1.11E−02	4.06E−02	3.55E−05	3.41E−02	1.70E−02	1.00E−03
F8	Mean	**−1.26E + 04**	**−**1.28E + 31	**−**3.18E + 03	**−**3.23E + 03	**−**9.50E + 03	**−1.26E + 04**	**−1.26E + 04**	**−**3.28E + 03	**−**4.06E + 03	NaN
	SD	4.12E−04	1.08E + 29	5.14E + 02	1.55E + 02	1.10E + 02	4.43E−02	1.57E−01	3.81E + 02	5.75E + 01	NaN
F9	Mean	**0.00E + 00**	8.29E + 01	4.10E + 01	**0.00E + 00**	2.76E + 01	**0.00E + 00**	**0.00E + 00**	1.95E + 01	7.05E + 00	1.45E + 01
	SD	0.00E + 00	6.91E + 00	1.11E + 01	0.00E + 00	2.94E + 00	0.00E + 00	0.00E + 00	1.23E + 01	1.60E + 00	1.03E + 01
F10	Mean	**8.88E−16**	2.08E−02	3.77E + 00	2.22E−11	5.51E + 00	**8.88E−16**	**8.88E−16**	4.62E−15	3.98E + 00	1.95E−15
	SD	0.00E + 00	4.39E−03	9.40E−01	7.62E−13	4.57E−01	0.00E + 00	0.00E + 00	7.94E−16	1.02E + 00	1.72E−15
F11	Mean	**0.00E + 00**	7.09E−02	1.51E−02	2.39E−11	4.23E + 00	**0.00E + 00**	**0.00E + 00**	1.23E−01	1.10E + 00	7.40E−04
	SD	0.00E + 00	3.51E−02	1.46E−02	7.99E−15	6.26E−01	0.00E + 00	0.00E + 00	5.93E−02	1.38E−01	2.30E−03
F12	Mean	2.17E−30	4.11E−03	2.09E−01	5.97E−01	4.45E−01	7.32E−04	2.13E−07	2.03E−01	1.99E + 00	**4.71E−32**
	SD	4.59E−31	1.91E−03	1.06E−01	1.14E−01	6.55E−01	2.74E−03	1.62E−06	5.84E−01	1.49E + 00	1.15E−47
F13	Mean	1.60E−30	6.82E−03	3.36E−01	2.92E + 00	3.26E + 01	7.24E−04	1.45E−06	3.30E−03	4.66E + 00	**1.35E−32**
	SD	2.09E−30	3.43E−03	6.06E−01	1.50E−01	1.60E + 01	6.22E−04	7.10E−12	5.20E−03	2.32E + 00	2.89E−48
F14	Mean	**9.98E−01**	**9.98E−01**	1.49E + 00	1.12E + 00	**9.98E−01**	**9.98E−01**	**9.98E−01**	1.25E + 00	9.98E−01	7.51E + 00
	SD	0.00E + 00	0.00E + 00	7.02E−01	3.15E−01	1.67E−08	2.70E−12	0.00E + 00	7.10E−01	1.75E−06	5.32E + 00
F15	Mean	1.74E−03	2.03E−03	4.36E−04	5.01E−04	5.55E−03	7.44E−03	**3.47E−04**	1.10E−03	4.86E−04	4.16E−03
	SD	1.12E−04	7.02E−04	4.07E−04	3.23E−04	3.17E−02	1.10E−02	1.11E−09	4.55E−04	1.46E−04	1.37E−03
F16	Mean	**−1.03E + 00**	**−1.03E + 00**	**−1.03E + 00**	**−**1.29E + 00	**−1.03E + 00**	**−1.03E + 00**	**−1.03E + 00**	**−1.03E + 00**	**−1.03E + 00**	**−1.03E + 00**
	SD	0.00E + 00	0.00E + 00	0.00E + 00	7.21E−01	5.12E−02	6.29E−12	0.00E + 00	2.28E−16	7.20E−17	0.00E + 00
F17	Mean	**3.98E−01**	**3.98E−01**	**3.98E−01**	4.05E−01	1.08E + 00	**3.98E−01**	**3.98E−01**	3.98E−01	3.98E−01	**3.98E−01**
	SD	0.00E + 00	0.00E + 00	0.00E + 00	1.42E−02	6.04E−01	5.73E−08	0.00E + 00	0.00E + 00	3.10E−14	0.00E + 00
F18	Mean	**3.00E + 00**	**3.00E + 00**	**3.00E + 00**	3.12E + 00	5.71E + 00	**3.00E + 00**	**3.00E + 00**	**3.00E + 00**	**3.00E + 00**	**3.00E + 00**
	SD	7.26E−16	5.34E−16	4.19E−16	2.02E−01	1.90E−03	9.01E−12	1.24E−06	9.34E−16	3.13E−15	0.00E + 00
F19	Mean	**−3.86E + 00**	**−3.86E + 00**	**−3.86E + 00**	**−**3.79E + 00	**−**3.85E + 00	**−3.86E + 00**	**−3.86E + 00**	**−3.86E + 00**	**−3.86E + 00**	**−3.86E + 00**
	SD	3.58E−11	9.36E−16	1.10E−03	9.30E−02	6.67E−04	7.37E−08	0.00E + 00	2.26E−15	3.28E−14	9.36E−16
F20	Mean	**−**2.71E + 00	**−3.32E + 00**	**−**3.29E + 00	**−**2.55E + 00	**−**2.84E + 00	**−**3.22E + 00	**−**3.79E + 00	**−**3.22E + 00	**−3.32E + 00**	**−**3.27E + 00
	SD	1.75E−01	1.97E−12	5.74E−02	3.71E−01	1.04E−05	4.36E−02	2.05E−03	4.73E−02	1.35E−04	6.14E−02
F21	Mean	**−1.01E + 01**	**−**9.59E + 00	**−**5.91E + 00	**−**4.80E + 00	**−**6.12E + 00	**−**1.02E + 01	**−**5.06E + 00	**−**6.89E + 00	**−**1.02E + 01	**−**7.17E + 00
	SD	1.87E−15	1.25E−02	3.73E + 00	9.52E−02	1.70E−02	3.43E−05	2.43E−06	3.73E + 00	1.37E−04	3.86E + 00
F22	Mean	**−1.04E + 01**	**−**1.02E + 01	**−**6.68E + 00	**−**4.70E + 00	**−**6.43E + 00	**−1.04E + 01**	**−**5.09E + 00	**−**7.99E + 00	**−1.04E + 01**	**−**9.64E + 00
	SD	5.92E−16	2.31E−02	3.94E + 00	1.46E−01	2.65E + 00	5.32E−05	8.60E−03	3.41E + 00	3.90E−03	2.42E + 00
F23	Mean	**−1.05E + 01**	**−**1.04E + 01	**−**9.23E + 00	**−**4.66E + 00	**−**4.92E + 00	**−1.05E + 01**	**−**5.13E + 00	**−**7.76E + 00	**−1.05E + 01**	**−**9.93E + 00
	SD	1.54E−02	2.11E−02	2.82E + 00	1.95E−01	2.39E + 00	6.40E−05	4.28E−03	3.53E + 00	1.35E−04	1.90E + 00
cec01	Mean	6.68E + 04	1.75E + 12	3.59E + 12	5.74E + 04	3.38E + 10	**4.19E + 04**	6.20E + 04	1.23E + 10	5.49E + 09	1.15E + 09
	SD	1.65E + 04	2.11E + 10	1.75E + 12	9.76E + 03	2.39E + 10	2.91E + 04	4.28E + 04	2.10E + 10	4.91E + 09	7.96E + 07
cec02	Mean	1.74E + 01	**1.73E + 01**	1.41E + 04	1.78E + 01	3.16E + 01	1.74E + 01	**1.73E + 01**	1.75E + 01	1.74E + 01	1.47E + 02
	SD	4.49E−01	3.74E−15	3.51E + 03	1.79E−01	1.21E + 01	3.63E−02	0.00E + 00	3.74E−03	1.21E−02	3.35E + 01
cec03	Mean	**1.27E + 01**	**1.27E + 01**	**1.27E + 01**	**1.27E + 01**	**1.27E + 01**	**1.27E + 01**	**1.27E + 01**	**1.27E + 01**	**1.27E + 01**	**1.27E + 01**
	SD	6.87E−08	1.97E−06	1.87E−05	4.42E−04	2.65E−07	4.16E−03	0.00E + 00	4.89E−06	9.93E−08	1.20E−03
cec04	Mean	9.45E + 01	1.64E + 03	3.75E + 03	1.61E + 04	4.91E + 04	**2.91E + 01**	4.84E + 01	8.16E + 01	1.25E + 02	2.75E + 01
	SD	3.30E + 01	3.88E + 02	2.09E + 02	7.38E + 03	2.29E + 04	1.13E + 01	0.00E + 00	1.14E + 02	3.55E + 01	4.98E + 00
cec05	Mean	2.25E + 00	3.18E + 00	3.28E + 00	4.80E + 00	3.60E + 00	2.31E + 00	2.46E + 00	2.30E + 00	1.49E + 00	**1.00E + 00**
	SD	2.23E−01	3.45E−01	4.99E−01	5.62E−01	1.80E + 00	1.27E−01	0.00E + 00	2.41E−01	1.11E−01	2.30E−03
cec06	Mean	9.08E + 00	1.35E + 01	1.13E + 01	1.17E + 01	1.19E + 01	**5.95E + 00**	8.36E + 00	4.91E + 00	7.49E + 00	1.14E + 01
	SD	7.81E−01	9.93E + 00	9.47E + 00	4.23E−01	6.14E + 00	8.50E−01	7.39E−01	2.09E + 00	9.36E−01	4.26E−01
cec07	Mean	2.75E + 02	2.98E + 03	1.30E + 03	8.38E + 02	3.04E + 03	2.95E + 02	3.56E + 02	4.51E + 02	**2.23E + 02**	2.85E + 02
	SD	7.96E + 01	1.24E + 02	4.70E + 02	1.78E + 02	1.11E + 02	1.85E + 02	3.30E + 02	2.37E + 02	9.45E + 01	5.01E + 02
cec08	Mean	**6.02E + 00**	6.26E + 00	6.22E + 00	6.96E + 00	9.92E + 00	6.06E + 00	6.20E + 00	6.02E + 00	6.06E + 00	6.93E + 00
	SD	5.94E−01	3.76E−01	8.63E−01	2.62E−01	8.31E + 00	1.01E−01	5.76E−01	6.25E−01	4.88E−01	1.66E−01
cec09	Mean	3.15E + 00	6.12E + 00	5.26E + 00	1.87E + 03	3.13E + 03	**2.37E + 00**	2.68E + 00	2.55E + 00	2.87E + 00	2.45E + 00
	SD	4.67E−01	4.12E + 00	5.74E−01	5.19E + 02	2.82E + 02	3.22E−01	6.29E−01	1.24E−01	1.48E−01	3.66E−02
cec10	Mean	**2.01E + 01**	2.04E + 01	2.09E + 01	2.04E + 01	1.83E + 02	**2.01E + 01**	**2.01E + 01**	**2.01E + 01**	**2.01E + 01**	**2.04E + 01**
	SD	1.15E−01	1.68E−01	1.37E−01	7.33E−02	5.30E + 01	1.61E−02	9.87E−02	8.42E−02	5.62E−02	1.24E−01

**Figure 5:**
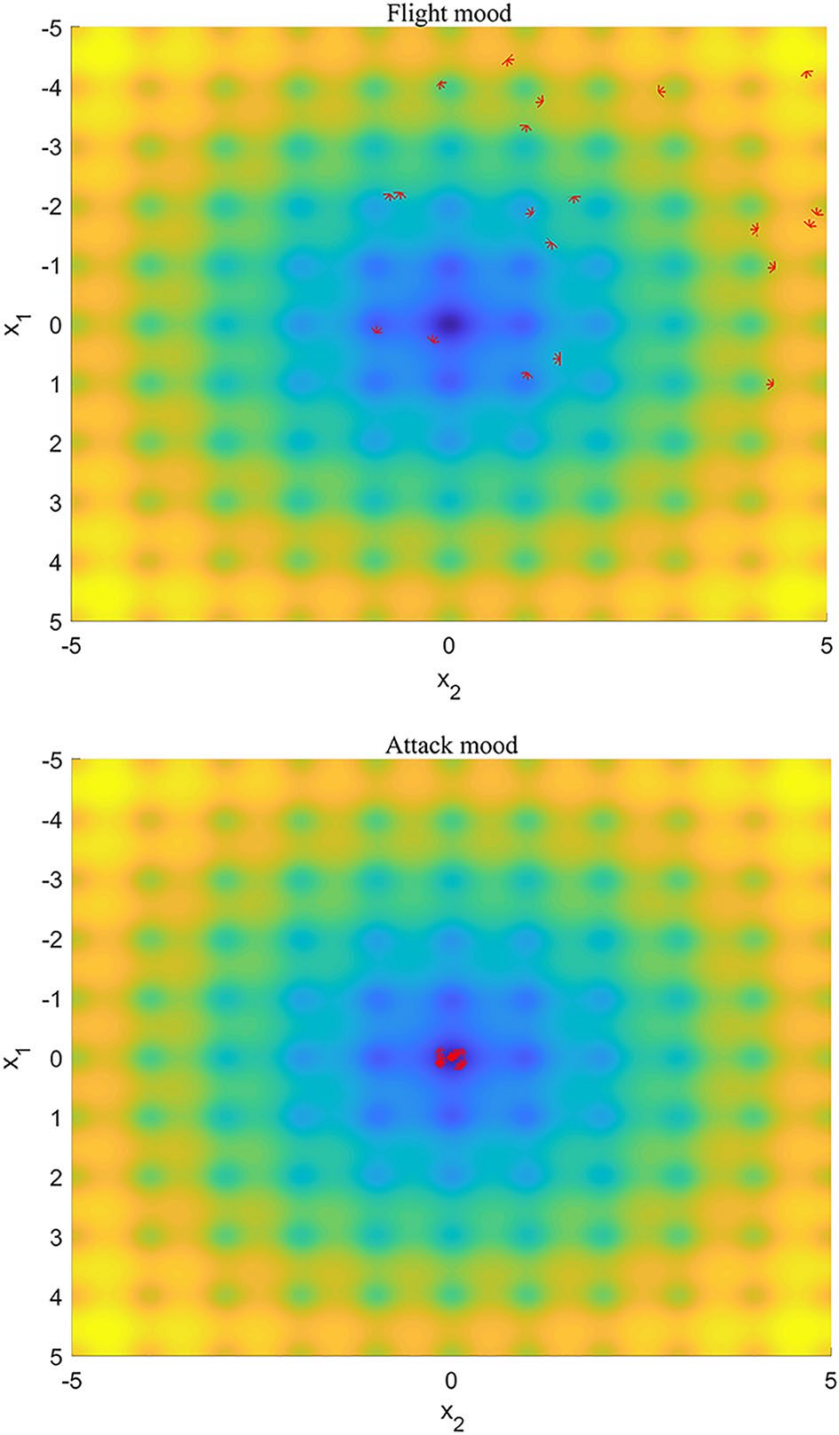
Aphids’ locations after 50 iterations in flight mood and their locations after 30 iterations in attack mood. The distribution of 20 aphids on a multimodal function after 30 iterations of flight mood and 20 iterations of the attack mood.

In addition, we use Fig. 5 to show the distribution of population on a multimodal function named Ackley's Function after 30 iterations of flight mood and 20 iterations of the attack mood. In the flight mood, individuals adaptively choose flight modes according to the crowding state among populations, thus maintaining the diversity of populations. In the attack mood, individuals are attracted by the host plants and gather near the optimal solution, which shows the strong exploration ability of the algorithm.

**Figure 6:**
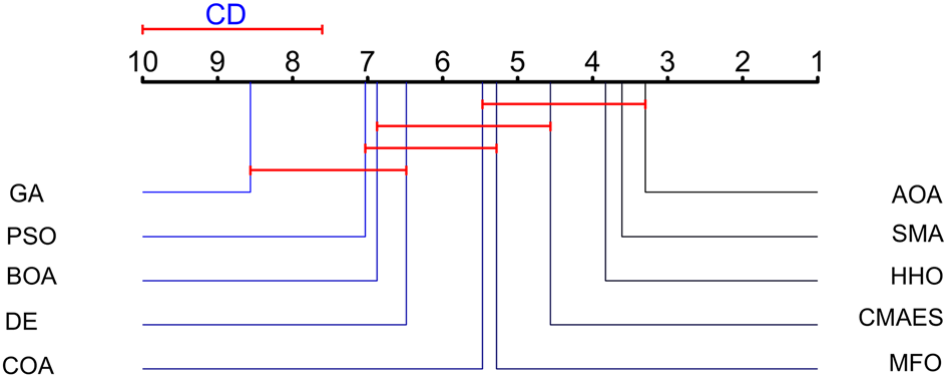
Plots of critical differences. Critical Differences plotting by post-hoc test.

To further demonstrate the significant difference between the proposed algorithm and other algorithms, we perform the Friedman test^46^ with α = 0.05 significance level, which is shown in Table 7. We list the average ranking of the results of the algorithms on all benchmark test functions with *p* < 0.05 in Table 7. It can be concluded from the table that AOA outperforms all the other algorithms. To further analyze the significance of paired differences, we chose the Nemenyi test as a post hoc analysis method and drew a critical difference plot to visualize the results. From Fig. 6, it can be concluded that AOA is comparable with SMA, HHO, CMAES, MFO and COA, while it is significantly better than DE, BOA, PSO, and GA.

**Table 7:** Mean rank and *p*-value of Friedman test. The mean ranking of all compared algorithms on test functions.

	Avg	Rank
AOA	3.2969	1
DE	6.4844	7
PSO	7.0313	9
BOA	6.875	8
GA	8.5625	10
SMA	3.6094	2
HHO	3.8281	3
MFO	5.2813	5
CAO	5.4688	6
CMAES	4.5625	2
*p*-value	9.49061E-19

**Figure 7:**
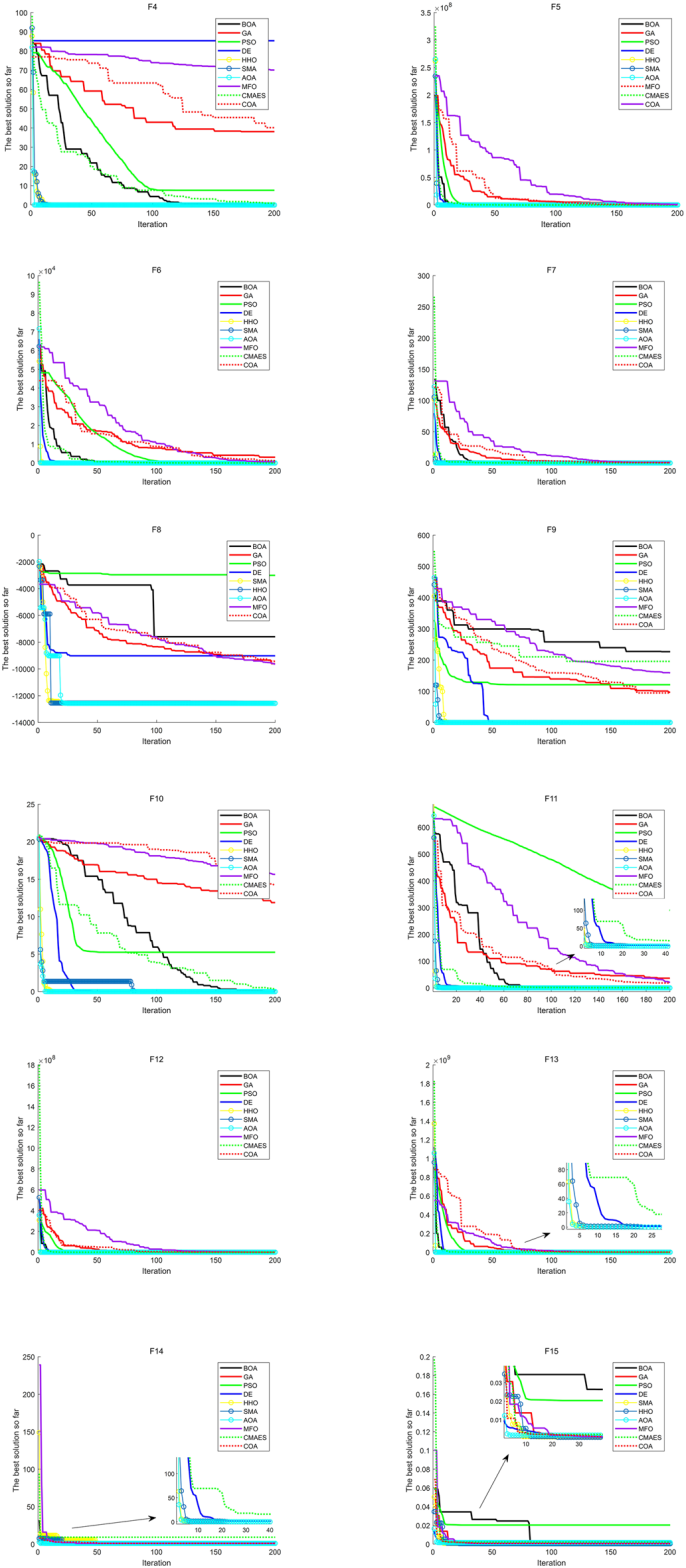
Convergence curves of benchmark functions. The figure shows the convergence speed of each algorithm by performing 200 iterations.

The convergence speed of an algorithm is also considered to be an important indicator for measuring algorithm performance. Figure 7 plots the convergence curves for 200 iterations of each algorithm. From Fig. 7, it can be seen that AOA outperforms most algorithms in the majority of test functions. As for F8, though the convergence speed of AOA is slower than that of other algorithms at the beginning of the iteration, its ability to find the global optimal solution at the later stage is almost the same as SMA and HHO. F16 is a multimodal function, and there are many local minima distributed in the domain of definition, which makes it more difficult for the algorithm to find the global minimum. Although SMA and HHO converge faster than AOA at the beginning, the accuracy is the same at later stage.

### Engineering problems

In this section, we solve two engineering design problems to test the capability of the proposed AOA algorithm and compare the result of AOA with that of other metaheuristic algorithms. These problems have several inequality constraints, which show the capability of AOA in solving constrained optimization problems. In this study, the constrained optimization problem is transformed into a series of unconstrained optimization problems to be solved by converting the constraints into penalty functions to be added to the objective function.

### Welded beam design

**Figure 8:**
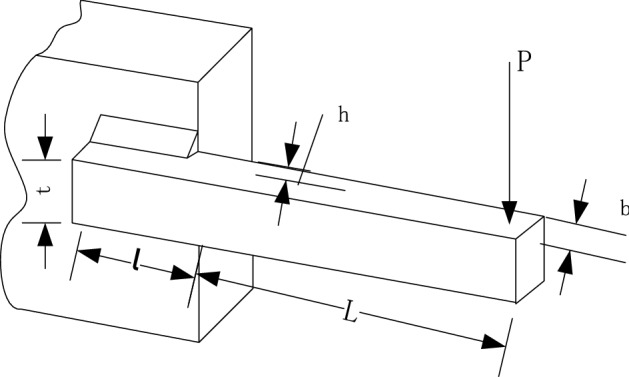
Welded beam problem. Model of welded beam.

The purpose of this problem is to minimize the cost of welded beam design under the condition of strength as shown in Fig. 8. In this problem, the thickness of weld (ℎ), length (*l*), height (*t*), and thickness of the bar (*b*) are selected as the design variables, and the cost of the welded beam is taken as the objective function. The mathematical formulation of this problem is as follows:

$$ {\text{Consider}}\;{\varvec{x}} = \left[ {h\, l\, t\, b} \right] = \left[ {x_{1} x_{2} x_{3} x_{4} } \right] $$

$$ {\text{Minimize}}\;f\left( {\varvec{x}} \right) = 1.10471x_{1}^{2} x_{2} + 0.04811x_{3} x_{4} \left( {14 + x_{2} } \right) $$

Subject to:

9 $$ g_{1} \left( {\varvec{x}} \right) = \tau \left( {\varvec{x}} \right) - 13600 \le 0 $$

10 $$ g_{2} \left( {\varvec{x}} \right) = \sigma \left( {\varvec{x}} \right) - 30000 \le 0 $$

11 $$ g_{3} \left( {\varvec{x}} \right) = x_{1} - x_{4} \le 0 $$

12 $$ g_{4} \left( {\varvec{x}} \right) = 0.10471(x_{1}^{2} ) + 0.04811x_{3} x_{4} \left( {14 + x_{2} } \right) - 5 \le 0 $$

13 $$ g_{5} \left( {\varvec{x}} \right) = \delta \left( x \right) - 0.25 \le 0 $$

14 $$ g_{6} \left( {\varvec{x}} \right) = 6000 - p_{c} \left( x \right) \le 0 $$

where

15 $$ \tau \left( {\varvec{x}} \right) = \sqrt {\left( {\tau^{\prime}} \right) + \left( {2\tau^{\prime}\tau^{\prime\prime}} \right)\frac{{x_{2} }}{2R} + \left( {\tau^{\prime\prime}} \right)^{2} } $$

16 $$ \tau^{\prime} = \frac{6000}{{\sqrt 2 x_{1} x_{2} }} $$

17 $$ \tau^{\prime\prime} = \frac{MR}{J} $$

18 $$ M = 6000\left( {14 + \frac{{x_{2} }}{2}} \right) $$

19 $$ R = \sqrt {\frac{{x_{2}^{2} }}{4} + \left( {\frac{{x_{1} + x_{3} }}{2}} \right)^{2} } $$

20 $$ J = 2\left\{ {x_{1} x_{2} \sqrt 2 \left[ {\frac{{x_{2}^{2} }}{12} + \left( {\frac{{x_{1} + x_{3} }}{2}} \right)^{2} } \right]} \right\} $$

21 $$ \sigma \left( {\varvec{x}} \right) = \frac{504000}{{x_{4} x_{3}^{2} }} $$

22 $$ \delta \left( {\varvec{x}} \right) = \frac{65856000}{{\left( {30 \times 10^{6} } \right)x_{4} x_{3}^{3} }} $$

23 $$ p_{{c}} \left( {\varvec{x}} \right) = \frac{{4.013\left( {30 \times 10^{6} } \right)\sqrt {\frac{{x_{3}^{2} x_{4}^{6} }}{36}} }}{196}\left( {1 - \frac{{x_{3} \sqrt {\frac{{30 \times 10^{6} }}{{4\left( {12 \times 10^{6} } \right)}}} }}{28}} \right) $$

Variable ranges are given as $0.1 \le x_{1} ,x_{4} \le 2$, $0.1 \le x_{2} ,x_{3} \le 10$.

The result of AOA is compared with DE, PSO, and GA as shown in Table 8. The bold values indicate the best one among all methods. The table demonstrates that AOA performs better than other algorithms in this engineering problem.

**Table 8:** Comparison of results for welded beam problem. Results of AOA and other different algorithms on welded beam problem.

Algorithm	Optimum variables				Optimum value
	*h*	*l*	*t*	*b*	
AOA	0.2056	3.9764	9.8395	0.2128	1.9965
PSO	0.2443	6.2059	8.3079	0.2453	2.3898
GA	0.2845	2.9364	7.9174	0.3462	2.4959
DE	0.2336	5.6579	9.6553	0.2766	2.8669

### Reliability analysis of bolt

**Figure 9:**
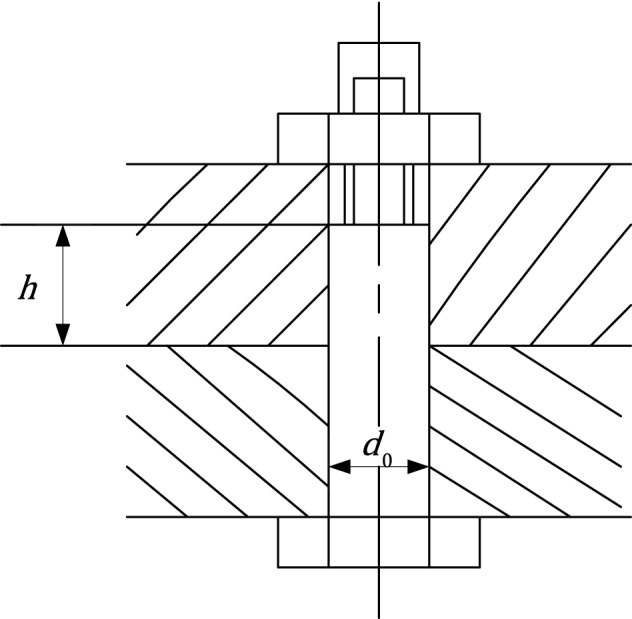
Bolt reliability analysis problem. Model of bolt.

The bolted connection of the rigid coupling is optimally designed under the condition of predetermined reliability R (set R ≥ 0.99), with the lightest bolt mass as the optimization target as shown in Fig. 9. The number of the limit bolt is 4 ≤ *n* ≤ 8, and the diameter of the bolt is 5 ≤ *d*_0_ ≤ 18 mm. The mathematical expression is as follows:

$$ {\text{Consider}}\;{\varvec{x}} = \left[ {n d_{0} } \right] $$

$$ {\text{Minimize}}\;f\left( {\varvec{x}} \right) = 2.6 \times 10^{ - 4} nd_{0}^{3} $$

Subject to

24 $$ g_{1} \left( {\varvec{x}} \right) = \frac{{200 - \frac{{24 \times 10^{4} }}{{\pi nd_{0}^{2} }}}}{{2.326\sqrt {14^{2} + \left( {\frac{{12 \times 10^{3} }}{{\pi nd_{0}^{2} }}} \right)^{2} } }} - 1 \ge 0 $$

25 $$ g_{2} \left( {\varvec{x}} \right) = \frac{{150 - \frac{{12 \times 10^{4} }}{{\pi nd_{0}^{2} }}}}{{2.326\sqrt {10.5^{2} + \left( {\frac{{6 \times 10^{3} }}{{\pi nd_{0}^{2} }}} \right)^{2} } }} - 1 \ge 0 $$

26 $$ g_{3} \left( {\varvec{x}} \right) = \frac{{280 - \frac{{7.5 \times 10^{4} }}{{\pi nd_{0}^{2} }}}}{{2.326\sqrt {25.2^{2} + \left( {\frac{{37.5 \times 10^{3} }}{{\pi nd_{0}^{2} }}} \right)^{2} } }} - 1 \ge 0 $$

27 $$ g_{4} \left( {\varvec{x}} \right) = \frac{n}{4} - 1 \ge 0 $$

28 $$ g_{5} \left( {\varvec{x}} \right) = 1 - \frac{n}{8} \ge 0 $$

29 $$ g_{6} \left( {\varvec{x}} \right) = \frac{{d_{0} }}{5} - 1 \ge 0 $$

30 $$ g_{7} \left( {\varvec{x}} \right) = 1 - \frac{{d_{0} }}{18} \ge 0 $$

The result of AOA is compared with DE, PSO, and GA as shown in Table 9. The bold values indicate the best one among all methods. As can be seen from the table, AOA outperforms all the other algorithms.

**Table 9:** Comparison of results for reliability analysis problem. Results of AOA and other different algorithms on reliability analysis problem.

Algorithm	Optimum variables		Optimum value
	*n*	*d*_0_	
AOA	7.8470	7.7437	0.94738
PSO	7.4601	7.9551	0.97646
GA	7.9536	10.0049	2.07096
DE	5.4063	9.4754	1.19582

## Conclusion

We proposed a new bio-inspiration based optimization algorithm called Aphid Optimization Algorithm (AOA) to solve optimization problems. The algorithm simulates the foraging process of aphids with wings, including the generation of winged aphids, flight mood, and attack mood. Concurrently, the corresponding optimization models have been presented according to the above phases. By comparing the performance of AOA with existing algorithms on 33 benchmark functions and two constrained engineering design problems, AOA algorithm has been shown its overall superiority. The presentation of AOA provides a new approach for solving optimization problems in the field of swarm intelligence optimization algorithms.

In future work, incorporating variation mechanisms and combinations with other algorithms will be considered to improve the performance of the algorithm. A future research direction also includes multi-objective AOA to solve multi-objective problems.

## Data Availability

All data generated or analyzed during this study are included in this article.
